# A 3D-Printed Ultra-Low Young’s Modulus β-Ti Alloy for Biomedical Applications

**DOI:** 10.3390/ma13122792

**Published:** 2020-06-20

**Authors:** Massimo Pellizzari, Alireza Jam, Matilde Tschon, Milena Fini, Carlo Lora, Matteo Benedetti

**Affiliations:** 1 Department of Industrial Engineering, University of Trento, 38123 Trento, Italy; matteo.benedetti@unitn.it; 2 Laboratory of Preclinical and Surgical Studies, IRCCS-Istituto Ortopedico Rizzoli, 40136 Bologna, Italy; matilde.tschon@ior.it (M.T.); milena.fini@ior.it (M.F.); 3 SISMA Spa, 36013 Piovene Rocchette (VI), Italy; clora@sisma.com

**Keywords:** 3D-printing, orthopaedic biomaterials, bone prosthesis, β-Titanium alloy, Young’s modulus, cytotoxicity

## Abstract

The metastable β-Ti21S alloy is evaluated as a potential candidate for biomedical parts. Near fully dense (99.75 ± 0.02%) samples are additively manufactured (that is, 3D-printed) by laser powder-bed fusion (L-PBF). In the as-built condition, the material consists of metastable β-phase only, with columnar grains oriented along the building direction. The material exhibits an extremely low Young’s modulus (52 ± 0.3 GPa), which was never reported for this type of alloy. The combination of good mechanical strength (σ_y0.2_ = 709 ± 6 MPa, ultimate tensile strength (UTS) = 831 ± 3 MPa) and high total elongation during tensile test (21% ± 1.2%) in the as-built state, that is, without any heat treatment, is close to that of the wrought alloy and comparable to that of heat treated Ti grade 5. The good biocompatibility attested by cytotoxicity tests confirms its great suitability for biomedical applications.

## 1. Introduction

Among metallic biomaterials for biomedical and specifically orthopaedic application, titanium and its alloys exhibit the most suitable characteristics as compared with stainless steels and Co-Cr alloys because of their high biocompatibility, specific strength, and corrosion resistance [1]. According to their phase constitution, Ti-alloys are classified into three main groups, namely, α, β, and α + β alloys. In essence, the microstructure depends on type and amount of alloying elements, as isomorphous α-phase stabilizers (Zr, Al, Sn, O, and Si), dissolved preferentially in the α phase, expand its phase field, while isomorphous β-phase stabilizers (H, Mo, W, and V), dissolved in the β phase, play the same role on the β-phase field [2]. Depending on the degree of alloying and thermomechanical processing path, it is possible to tune the balance of α and β phases, which permits to tailor properties like strength, toughness, and fatigue resistance.

Fully α alloys have important strength limitations owing to reactions occurring at high temperatures, during hot forming. Therefore, the development of Ti-alloys has been mainly focused on α + β ones. Until recently, the guidelines followed for the introduction of biomaterials for hard tissue substitution in orthopedic applications have involved the adaptation of existing materials, as exemplified by the use of Ti–6Al–4V extra-low interstitial (ELI), an alloy originally designed for aerospace applications. Besides Ti–6Al–4V ELI (ASTM F 136), only Ti–6Al–7Nb (ASTM F 1295) has been standardized for biomaterials in ASTM. However, one of the major limitations of α + β alloys is given by their relatively high Young’s modulus E, being comprised between 110 and 120 GPa [1]. The resulting stiffness mismatch between bony tissue (Young’s modulus is equal to 10 ÷ 20 GPa and 0.1 ÷ 1 GPa for cortical and cancellous bone, respectively) and implant causes stress shielding and bone resorption [3,4].

In view of the lower elastic modulus of body center cubic (bcc) β-phase (50 GPa < E < 100 GPa) as compared with the hexagonal close-packed (hcp) α-one, as well as owing to their good mechanical properties, excellent corrosion resistance, and biocompatibility, β-Ti alloys have been recently proposed as a valid alternative to α + β ones [5,6]. For this purpose, several alloying systems were explored in the past to confer adequate mechanical properties. To this regard, Mo-rich grades like Ti–12Mo–6Zr–2Fe (ASTM F1813) and Ti–15Mo–2.8Nb–0.2Si–0.28O with tensile strength >1000 MPa, total elongation >15%, and Young’s modulus around 80 GPa were developed for orthopaedic applications [3,4]. Among Nb-rich grades, Ti–13Nb–13Zr is worth mentioning because of the good property portfolio, even if precipitation hardening alloys as Ti–29Nb–13Ta–4.6Zr and Ti–16Nb–13Ta–4Mo have been proposed in the literature [7,8]. Materials used in biomedical applications must exhibit a high cycle fatigue strength. The results reported in the literature evidence a broad spectrum of fatigue strength for biomedical Ti alloys, ranging from 265 to 816 MPa [5]. This large variety can be mainly ascribed to the wide range of microstructural options (e.g., fully lamellar, bi-modal, pancake [9]) along with the possibility of hardening the metastable β-matrix through precipitation of fine particles of α-phase [1]. In this way, it is possible to achieve fatigue properties even superior to those of α + β alloys [10].

From a biological point of view, the compatibility and osseointegration of an implant in the surrounding living tissues can be seriously hindered by the release of metallic ions into the human body, causing sensitization, irritation, and inflammation. From a recent systematic review, metallic wear debris particles are responsible for upregulating the production of several pro-inflammatory cytokines, chemokines, and matrix metalloproteases that induce chronic inflammation, tissue fibrosis, and osteoclasts activation at the bone–implant interface [11]. Osteoclasts and osteoclastogenensis determine a progressive bone deterioration and enhance the osteolysis progress that can compromise the implant stability, leading to implant loosening [12,13,14].

Another limitation in the use of the gold standard Ti–6Al–4V ELI stems from its content of potentially cytotoxic alloying elements, namely Al and especially V [15]. There is hence a considerable interest in developing new Ti-alloy formulations without the addition of cytotoxic elements. In the context of β-Ti, which is the focus of the present paper, it is crucial to avoid the use of V as a β-stabilizing element [16]. For instance, Ti–15Mo–5Zr–3Al has been claimed to be a good candidate for biomedical implants, mainly thanks to the relatively low Young’s modulus (80 GPa), the good ductility (25%), and formability associated to the bcc structure along with adequate yield strength (830 MPa) [5]. It is well documented that this alloy can experience long-term exposure in the human body and that the crystal structure of the cast alloys changes from α′→α″→β, increasing the Mo content from 6 wt.% to 20 wt.% [17]. Ti–15Mo–2.7Nb–3Al–0.2Si grade can provide the same properties of Ti–6Al–4V for biomedical as well as for aerospace applications [18].

In designing new β-Ti alloy systems, the scenario depicted so far is further complicated by the growing interest towards fabricating biomedical prosthetic implants through additive manufacturing (AM) techniques [19]. AM will prospectively allow tailoring a specific implant to the patient and producing it on demand, with large savings on time and costs [20]. AM is gaining increasing interest owing to the possibility of producing orthopaedic implants with functionally graded open-cell porous metals [21]. Their purpose is to mimic the complex structure of the bone with the aim to increase the implant osseointegration [22]. The main advantages of porous materials are the reduction of the elastic modulus mismatch between bone and implant alloy, alleviating the stress shielding effect and improved implant morphology, providing biological anchorage for tissue in-growth [23].

Finding formulations of β-Ti alloys suitable to be additively manufactured is thus of vital importance for the current biomedical research. The attention has been focused so far on the addition of transition/refractory β stabilizing metals. Wang [24] investigated the effect of Nb content on the β phase stability of an additive manufactured (AMed) Ti–Nb system. Some other researchers [25,26] have compared AM of the Ti–Nb system via other production methods, such as hot pressing and hot forging. In particular, Zhou et al. [26] reported that the microstructure, properties, and phase formation are greatly influenced by the production method. Fischer and Schwab [27,28] manufactured Ti–26Nb and Ti–45Nb with mixed and pre-alloyed powder to achieve the β phase. Trabecular structure made up of β-alloys Ti–24Nb–4Zr–8Sn and Ti–30Nb–5Ta–3Zr were investigated in [29,30], showing, however, that the footprint of α precipitation was not erased entirely. Tantalum (Ta) has been introduced even if it is a rare-earth and expensive metal, and 50 wt.% thereof is necessary to fully stabilize the β-phase in high cooling rate solidification [31]. Recently, it was found that Ti–15Ta–xZr might have even better performances [32]. Ti–5Al–5V–5Mo–3Cr alloy was designed to meet aerospace demands [33]. However, its biocompatibility is doubtful owing to the presence of V and the fact that no cytotoxicity test was done. Some authors have attempted to address the drawbacks of the aforementioned elements by introducing Mo as the main alloying element. Vrancken et al. [34] performed considerable research on the AM production of β-Ti alloys. By introducing 10 wt.% Mo to Ti–6Al–4V ELI powder, the β→α′ martensitic transformation was suppressed, and a metastable β-structure could be achieved. Indeed, owing to the limited chemical homogeneity of the liquid inside the melting pool, a heat treatment was required to reduce the segregation of this element in the microstructure. The Young’s modulus (73 GPa) was lower than that of α + β alloys, but still much higher than that of the human bone (20 GPa). Nan Kang et. al. produced Ti–7.5Mo samples by selective laser melting, showing that the Mo content is not high enough to obtain a fully β-structure and a significant improvement in mechanical properties [35,36].

From the above discussion, it is clear that another AM-related advantage of β- over α + β Ti-alloys resides in the possibility of suppressing the martensitic transformation in suitably formulated β-Ti alloys. Indeed, the high cooling rates typical of the laser powder bed fusion (L-PBF) AM process lead to the formation of a brittle and soft α′ phase in α + β alloys [37]. In addition, the volumetric expansion associated with the martensitic transformation is responsible for undesired residual stresses and distortions [38]. For this reason, α + β alloys necessitate a heat treatment before removing the part from the L-PBF building platform either above or below the β-transus to obtain a fine acicular or a coarse lamellar microstructure, respectively [39,40]. This, however, represents an additional and delicate manufacturing step, which must be carried out carefully to avoid detrimental oxygen pickup [41]. Unfortunately, vacuum furnaces are not always within the reach of any biomedical manufacturer.

With this in mind, the present work is aimed at identifying a Ti-alloy formulation suitable to be manufactured via L-PBF without the necessity of any post-sintering treatment. In particular, it explores the potential use of the Ti–15Mo–2.7Nb–3Al–0.2Si alloy (β-Ti21S, 21 wt.% of alloying additions, including Silicon) for biomedical applications. Through microstructural, mechanical, and cytotoxicity analyses, we will show that this material exhibits (i) an unprecedented ultra-low elastic modulus, prospectively beneficial to the mechanical compatibility with the bone; (ii) an improved cytocompatibility owing to the lack of Vanadium; and (iii) the absence of the martensitic transformation responsible for hard and brittle solidification structures.

## 2. Materials and Methods

A prealloyed β-Ti21S alloy (GKN Hoeganaes Corporation, Cinnaminson, NJ, USA, D10 = 25 μm, D50 = 41 μm, D90 = 60 μm) produced by plasma-atomization was used. The chemical composition (wt.%) of the powder is 14.6% Mo, 2.8% Al, 2.8% Nb and 0.3% Si, 0.11% O, and 0.004% N, Ti balance.

Cylindrical samples (D = 4 mm, H = 10 mm) were fabricated with the main axis parallel to the building direction using an L-PBF machine model MYSINT100 (SISMA SPA, Piovene Rocchette, Italy) with a laser spot of 55 μm. The machine has an in-house developed building platform of 100 mm diameter and a 200 W fiber laser. Process parameters were optimized to achieve maximum density; the laser heat input was kept between 40 J/mm^3^ and 90 J/mm^3^. An XY alternate scan strategy was applied. In order to prevent oxygen pick-up, an argon atmosphere was used, reaching a 100 ppm O_2_ content inside the chamber. The process layer thickness was set to 20 µm. Density was measured by Archimedes’ principle according to ASTM standard on 10 mm diameter samples.

Tensile tests were carried out according to ASTM E8M at a strain rate of 1 mm/min on dogbone cylindrical specimens with 15 mm gage length and 5 mm diameter using a universal servohydraulic testing machine (model 8516, Instron, Norwood, MA, USA). Strains were measured using an axial extensometer with a 12.5 mm gauge length. Yield stress, Young’s modulus, and fracture strain were determined according to ASTM E 111; the average values and standard deviations were calculated considering at least three samples. Samples were tested parallel to the building direction and did not undergo any finishing step after L-PBF. The HV0.1 hardness was measured with a micro hardness tester (model FM-310, Future Tech, Kawasaki, Japan), according to ASTM E92, taking five measurements for each sample and reporting the average value from the top and lateral surfaces. In order to explore possible anisotropy in the material mechanical response, three cubic samples of 8 mm side were extracted from the terminal part of the tensile specimens and tested under compression along the three directions x, y, and z. Particular care was taken to cut the sample faces in order to properly align the sample with the loading direction. Compressions tests were carried out under stroke control with a strain rate of of 1 mm/min. Tests were stopped at 12% strain owing to the achievement of the load capacity of the testing machine. The compression axial strain was measured using an Instron LVDT, and the same machine used for the tensile tests.

The microstructural characterization of as-built samples was carried out by optical microscope (model Axiophot, Carl Zeiss EL-Einsatz, Jena, Germany) and scanning electron microscopy (SEM, model JSM-IT300LV, Jeol, Tokyo, Japan). The phase constitution was determined by X-ray diffraction (XRD) using a Co radiation (λ = 0.17889 nm), in-house developed machine in X-ray Lab of industrial engineering department, Trento, Italy. Texture and crystallographic orientation for taking out pole figure were evaluated by X-ray with (1) microfocus 50 W Cu source, 2D beam optic; (2) detctris eiger 1M 2S hybrid pixel detector; and (3) four circle huber goniometer. Both X-ray measurements were carried out on metallographic cross sections parallel and perpendicular to the building directions.

In vitro cytotoxicity was determined according to EN ISO 10993-5: 2009 for the β-Ti21S (test) and Ti–6Al–4V ELI (reference) samples [42]. MG63 human osteosarcoma cell line (Cell bank IRCCS San Martino IST, Genova, Italy) was used. Cells were thawed and expanded in a 75 mL flask using Dulbecco’s modified Eagle’s medium (DMEM, Merck KGaA, Darmstadt, Germany) with 10% foetal bovine serum (Euroclone S.p.A, Pero, Italy) and 100 IU/mL penicillin–100 μg/mL streptomycin (Gibco, Merck KGaA, Darmstadt, Germany) in standard culture conditions (37 °C in 5% CO_2_ humidified atmosphere). Cells were seeded at the concentration of 2 × 10^4^ cells/cm^2^ in multiwell plates with test and reference materials and appropriate negative (CTR−: negative ConTRol, cells without materials) and positive (CTR+: positive ConTRol, cells in presence of a known cytotoxic agent, 0.5% phenol solution in DMEM) controls were run concomitantly.

Plates were incubated for 24 h, at 37 °C in 5% CO_2_ atmosphere. Thereafter, cell viability assay, Neutral red, and Phallodin stainings were performed and supernatants collected for the measurement of lactate dehydrogenase (LDH) release.

Cell viability was evaluated by adding Alamar Blue Cell Viability Reagent (Thermo Fisher Scientific, Waltham, MA, USA) to the fresh medium; viable cells internalize and reduct non-fluorescent Resazurin to fluorescent Resorufin. After 3.5 h of incubation, fluorescence was red at 530ex–590em nm wavelengths by a micro plate reader (VICTOR X2030, Perkin Elmer, Italy) and expressed as a percentage of negative controls. Samples with cell viability below 70% were considered cytotoxic, as indicated in the ISO 10993 standard.

LDH release was measured by an enzyme-kinetic cytotoxicity detection kit (Roche Diagnostics Spa, Monza, Italy). Briefly, 100 μL of reagent was added to 100 μL of cell supernatant in a 96-well plate; after 30 min of incubation at room temperature in the dark, optical density (OD) were quantified by spectrophotometer at 490/655 nm. Cytotoxicity was calculated as follows (Equation (1)):

(1) $$\mathrm{Cytotoxicity}\;(\%)=\frac{\mathrm{OD}\;\mathrm{test}-\mathrm{OD}\;\mathrm{CTR}-}{\mathrm{OD}\;\mathrm{CTR}+-\mathrm{OD}\;\mathrm{CTR}-}\times 100$$

Neutral Red staining and quantification were performed by the in vitro toxicology assay kit (Merck KGaA, Darmstadt, Germany). Briefly, a 0.033% solution of the reagent in culture medium was added to all wells at the end of the experimental time for a further 90 min. Cultures were examined by light microscopy for the evaluation of cell morphology and images were taken (inverted microscope equipped with a Nikon digital camera model Eclipse, Melville, NY, USA). Then, the dye was solubilized by adding 1% acetic acid in 50% ethanol under gentle stirring in a shaker for 10 min. Absorbance was measured at a wavelength of 540 nm. Neutral red uptake was expressed as the percentage of negative controls.

Phalloidin staining was performed after cell fixation in a solution of 4% paraformaldehyde in phosphate buffered solution (PBS) for 15 min at 37 °C, permeabilization in 0.5% Triton X-100 for 15 min, and extensive washing steps in PBS. An Fluorescein Isothiocyanate -conjugate phalloidin solution (Merck KGaA, Darmstadt, Germany) 1:100 in PBS was added for 30 min at 37 °C and, after washing in PBS, samples were observed by fluorescence microscope (Nikon Eclipse, Nikon, Moncalieri, Italy).

Statistical evaluation of biomedical data was performed using the software v.23 package SPSS/PC + StatisticsTM 25.0 (SPSS Inc., Chicago, IL, USA). Data are reported as mean ± standard deviations (SD) at a significance level of *p* < 0.05 of three replicates. Data did not show a normal distribution and homogeneity of variance (Levene test), and thus a non-parametric analysis was carried out using Kruskal–Wallis followed by the Mann–Whitney U test to compare materials and controls.

## 3. Results and Discussion

### 3.1. Microstructure

**Figure 1:**
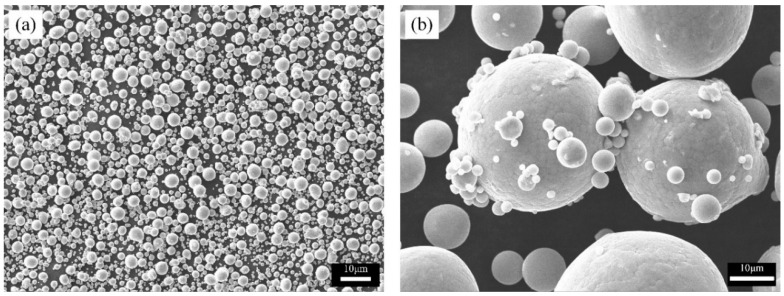
(**a**) General and (**b**) high magnification views of β-Ti21S powder (scanning electron microscopy, SEM).

A prealloyed β-Ti21S alloy displays a spherical particles shape (Figure 1). The particle size distribution evidences two distinct peaks, the first one centered around 10 μm related to satellite particles (Figure 1b), and the second one around 41 μm related to the powders major fraction.

**Figure 2:**
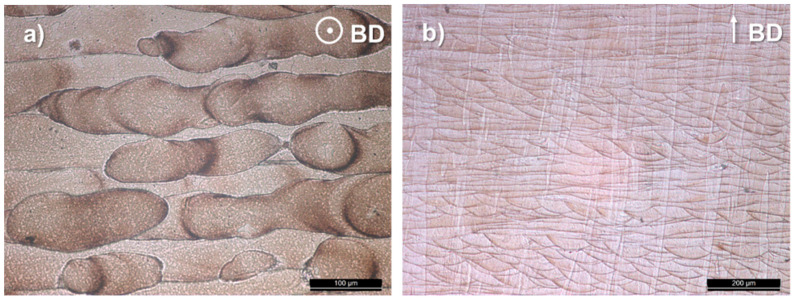
Top (**a**) and cross section (**b**) views of as-built β-Ti21S (optical microscope). BD, building direction.

The top and a cross sectional view of the microstructure highlight the achievement of a near fully dense material showing a columnar structure oriented along the building direction (Figure 2).

**Figure 3:**
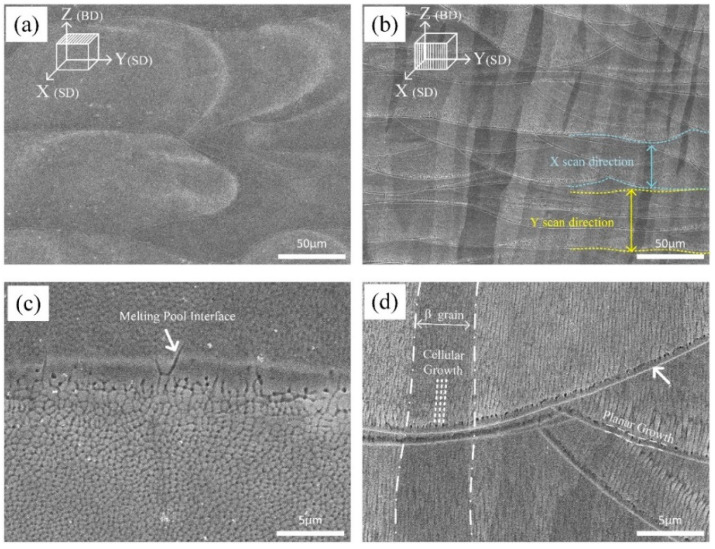
Low (**a**,**b**) and high (**c**,**d**) magnification SEM views from the top (**a**,**c**) and cross section (**b**,**d**) AB microstructure, columnar β grains growth were formed along the building direction (BD). Elongated cells grew in both X and Y scan directions (SD), and melting pool interface is indicated by the arrow.

SEM micrographs taken at higher magnification emphasize the traces of melting pools, which outline the alternate laser scan strategy used for the fabrication of samples (Figure 3a,b).

The epitaxial growth of β grain takes place along the heat flow direction and, according to previous works, arises owing to partial remelting of previously consolidated layers and extends up to several millimeters in length [43]. The average width of the β grains is 69 µm, which is close to the hatch spacing.

The solidification structure and particularly the grain orientation are influenced by the local heat flow direction [44], which is almost parallel to the building direction. Moreover, SEM micrographs taken at higher magnification evidence that the structure is planar at melt pool boundary, turning into cellular 0.5–1 µm inside the pool region (Figure 3c). The destabilization of the planar solidification front is owing to the establishment of constitutional undercooling and in particular to the decreasing temperature gradient inside the liquid ($T_{L}^{\prime }$) ahead of the solid/liquid interface. When $T_{L}^{\prime }$ becomes lower than the critical gradient ($T_{c}^{\prime }$), Equation (2), the planar to cellular transition may occur [45].

(2) $$T_{c}^{\prime }=\frac{T_{\mathrm{liq}}-T_{\mathrm{sol}}}{D/v}$$

T_liq_ = liquidus temperature;
T_sol_ = solidus temperature;
D = solute diffusivity in the liquid;
v = solidification speed.

In 15% Mo β-Ti21S alloy, this event is favoured by the large solidification range (T_liq_ − T_sol_) as well as by the very high solidification speed during the L-PBF process. A planar solidification front is observed instead in Ti–6Al–4V, showing a much narrower freezing range [6].

Inside each columnar grain, the cellular substructure shows an intercellular spacing of less than 0.6 µm (Figure 3d). The cells growth direction is near-vertically oriented, that is, along the temperature gradient, towards the top melt pool center. It closely follows the laser flow direction, that is, if the laser beam is moved from left to right, the grains are oriented rightwards (Figure 2b and Figure 3b).

### 3.2. Phase Constitution and Texture

**Figure 4:**
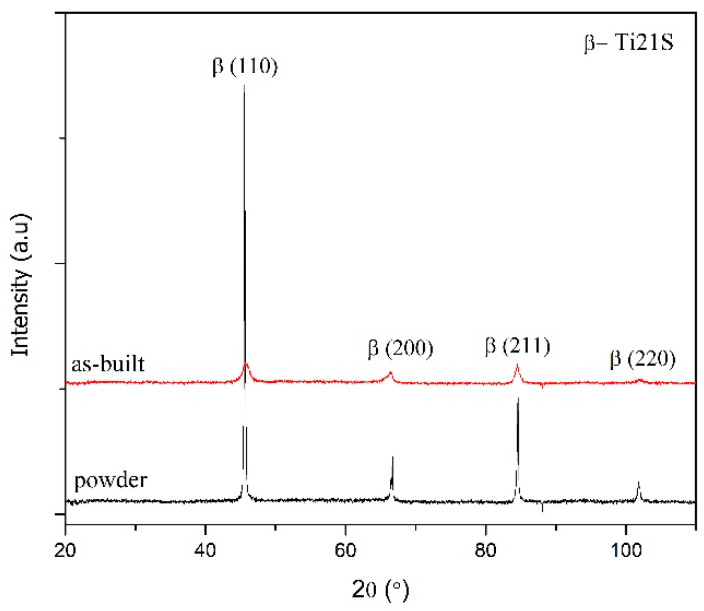
XRD patterns of initial powder and as-built β-Ti21S, fully stabilized β is the main phase in this system.

XRD analysis confirms that the as-built alloy is constituted by a single metastable β phase (Figure 4). Traces of neither α nor α′-martensite can be detected. Comparing the powder and as-built spectra, the peaks show a change in relative intensity, which is representative of the crystallographic texture in the as-built alloy. For approving this claim, we tried to get X-ray patterns from different orientations.

**Figure 5:**
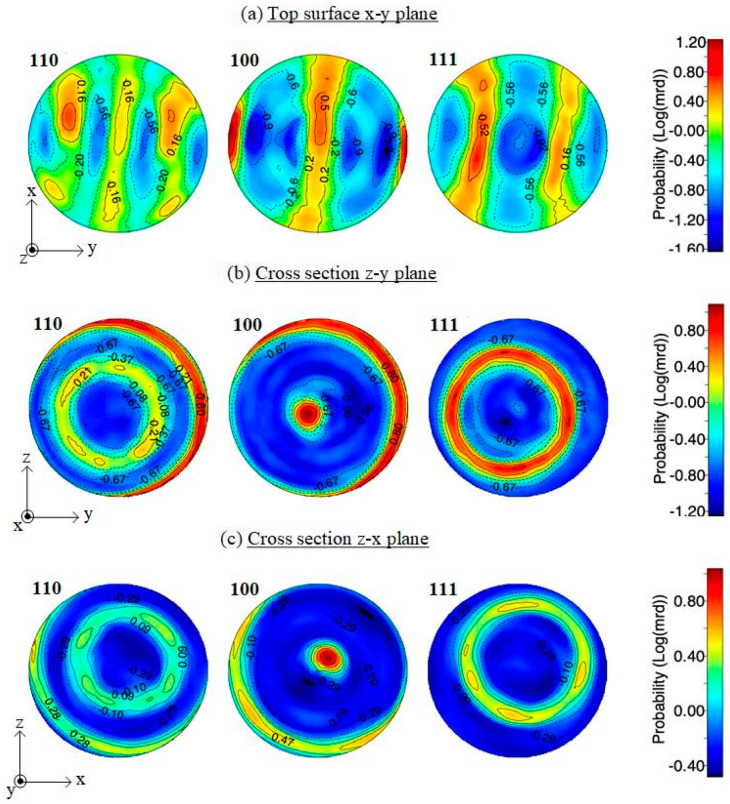
Pole figures were taken from x–y, z–y, and z–x planes, indicating <100> texture in parallels planes to the building direction Z.

Pole figures for the <110>, <100>, and <111> orientation from the top and cross sections are depicted in Figure 5. A preferential crystal growth orientation <100> is observed in the building direction Z in both the z–y and z–x planes, in agreement with the results obtained previously for different β-Ti alloys [34,43]. Ring texture pole figure in crystallographic orientation confirms a preferential <100> type growth in building direction (z), as well as in the x and y directions. This preferred orientation can be considered a potential source of anisotropy, which will plausibly result in mechanical properties changing along different directions.

### 3.3. Mechanical Properties

**Figure 6:**
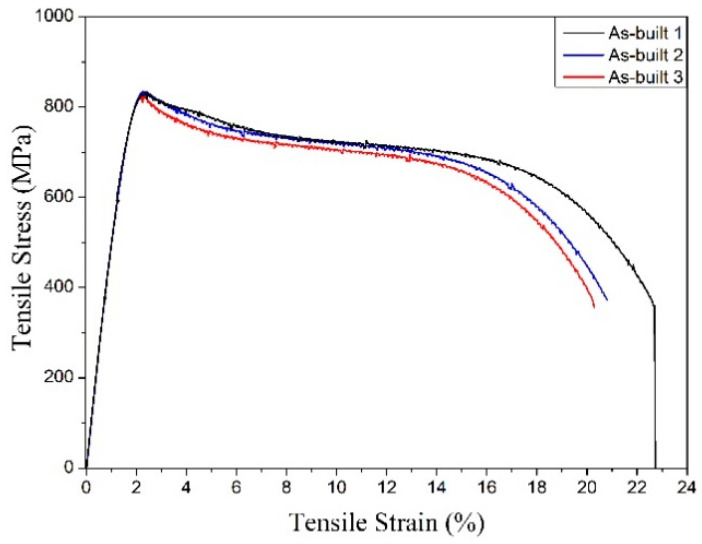
Stress–strain curves of the β-Ti21S produced by L-PBF under tensile loading along the z-direction.

The mechanical behavior of this β-Ti alloy is described by the engineering stress–strain curve showing an initial linear elastic region followed by a very intense work hardening (Figure 6). The principal results of the tensile tests are listed in Table 1 and compared with data found in the literature for Ti-alloys. Where available, the mechanical properties are indicated as mean ± standard deviation. Looking at Figure 6, it can be noted that the three replicated tensile tests lead to stress–strain curves exhibiting a flow softening resulting in a marked stress drop. This behavior, recently reviewed in [46], has been ascribed to the planar inhomogeneous plastic flow aided by localized adiabatic temperature rise. This is supported by the formation of intense planar slip bands, which originate from the relatively easy shearing of a thermal ω phase precipitates as well as their dissolution [47].

As a result, the mechanical strength is slightly lower than that reported for the same wrought alloy (sheet 0.56 mm) in the solution heat treated condition [47] (Table 1), but the fracture elongation is higher. As expected, the strength is much lower than that of annealed α + β AM–Ti–6Al–4V [39,48,49], but the result is worthy of attention, considering that the present figures were obtained without heat treatment. On the other hand, the high fracture elongation provides the condition for avoiding the heat treatment after additive manufacturing; preliminary results using cantilever beam samples confirm that samples undergo very limited distortion compared with standard α + β AM–Ti–6Al–4V and that any thermal stress during laser processing could be accommodated. The elastic part of the stress–strain curves depicted in Figure 6 is affected by very low scatter, as also confirmed by the low standard deviation of the elastic modulus reported in Table 1. A fundamental result of this work is the extremely low value of the Young’s, namely 52 ± 0.3 GPa, that is, about half of that of Ti–6Al–4V, which helps to prevent stress-shielding, maintaining at the same time an acceptable yield stress even higher than that of Ti–Na–Ta–Zr (TNTZ) β-type alloys. Comparing this result with those reported in the existing literature, it is evident that the dispersion band affecting this material property is clearly distinct from that displayed by the reported literature data. Even considering a very conservative 6sigma approach, the dispersion band of the Young’s modulus of the present material is not overlapped with that of the comparison variants. This gives a statistically robust proof that the material under investigation displays the lowest Young’s modulus attested so far in the literature for Ti-alloys.

**Figure 7:**
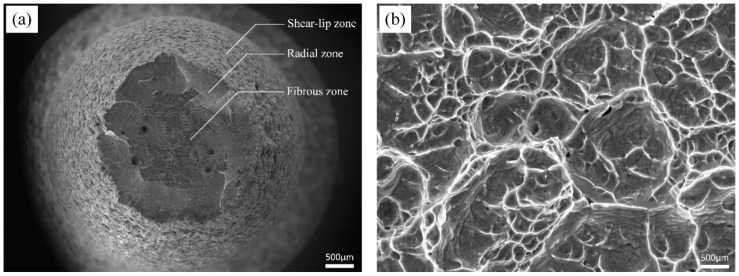
(**a**) Tensile fractography of β-Ti21S shows three zones as ductile manner. (**b**) Detail illustrating the fibrous zone.

Figure 7 displays the typical fracture surface of the tensile sample. Cavities correspond to cup-cone behavior, that is, the specimen is fractured in a ductile manner; three distinct zones as ductile characteristic fracture are pointed out in Figure 7a. 

In the fibrous zone near the center (Figure 7b), dimples with different sizes are shown. It can be inferred that a large amount of plastic deformation, and hence energy, will be required to induce fracture, as confirmed by the high fracture elongation of this alloy.

**Figure 8:**
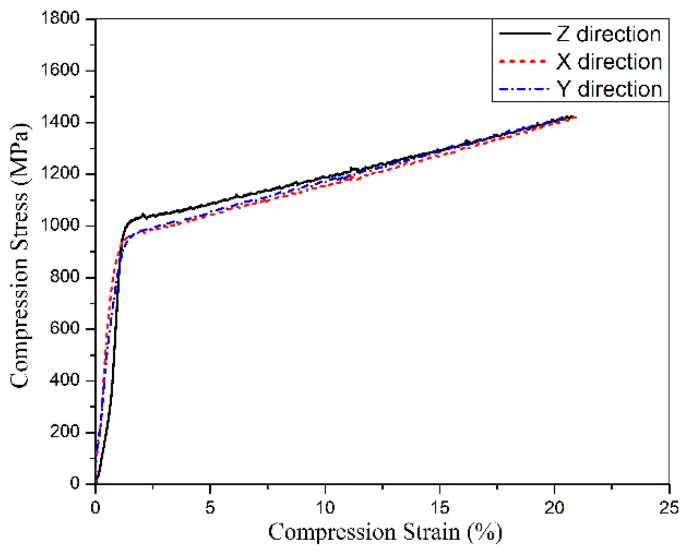
Stress–strain curves of the β-Ti21S produced by L-PBF under compression loading along the z-direction.

The mechanical anisotropy in β-Ti alloys is the result of a complex set of factors including texture and chemical composition, as well as microstructural characteristics like grain size, morphology, the presence of any α phase within the parent β matrix, extent of dynamic recrystallization in the case of hot deformation, and formation of in-grain shear bands in the deformed state [51]. Figure 8 shows the compression stress–strain curves recorded along three different directions. A first interesting difference with respect to the tensile test curves is the steady strain hardening after yielding. A second important result is the different elastic modulus along the x (64 ± 0.7 GPa), y (61 ± 0.9 GPa), and z (52 ± 0.5 GPa) directions. As expected, the major difference is observed along the building direction, showing the minimum value, about 15–19% lower those in the other two directions. On the other hand, the compressive strength is slightly higher along z. The noteworthy gap, which is defined as an anisotropic phenomenon, is likely owing to the specific ring texture, which has been discussed before. This anisotropy effect is also confirmed by the different hardness measured on the top (280 ± 2 HV0.1) and lateral surfaces (298 ± 3 HV0.1) of cylindrical samples.

A first source of anisotropy is the microstructure produced by the layer-wise nature of the AM process; mechanical properties are influenced by the orientation of the melt pool boundaries with respect to that of the applied stress. This is eventually emphasized by the presence of local defects like lack of fusion. Moreover, reheating caused by each new pass in the previous layer also introduces microstructural anisotropy, which will depend on the heat flow direction. One more important source of anisotropy is the columnar grains, aligned parallelly to the build direction. This effect was previously claimed to be the main cause of mechanical properties anisotropy in Ti–6Al–4V produced by AM, particularly strength and fracture elongation [52]. However, alterations in the linear elastic behaviour have been investigated in far less detail and the few existing studies are not in agreement. Previous studies [53,54] did not evidence any dependency of the Young’s modulus on the polar angle. On the basis of the results of Hitzler [55], however, important deviations in the elastic modulus were evidenced for 316 stainless steel. The nature of these deviations has not been explained, on a theoretical basis, but it is plausible that they are related to the texture and different bonding strength along different crystallographic directions (Figure 5).

### 3.4. In Vitro Cytotoxicity

**Figure 9:**
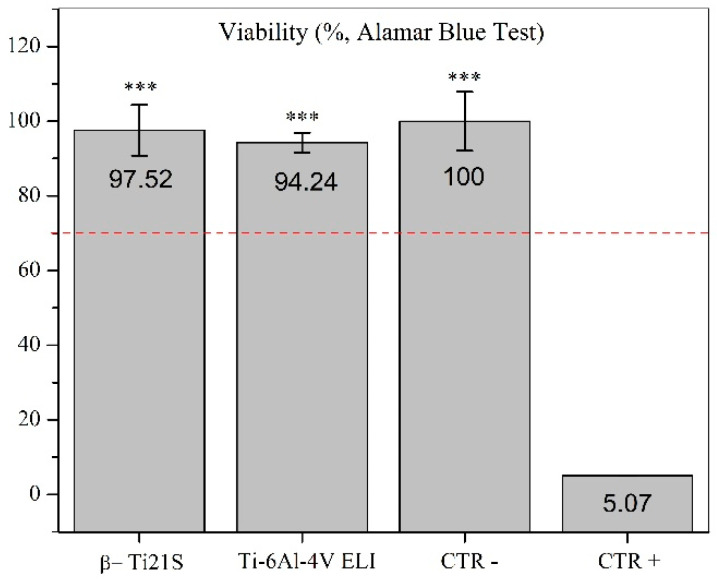
Viability results of the β-Ti21S (test), Ti–6Al–4V ELI (reference), and CTR− (cells without material) and CTR+ (cells with a cytotoxic substance) controls measured by Alamar Blue test and expressed as a percentage of CTR−. Red-dashed line represents 70% of negative control, which is a cut-off line between cytotoxic and non cytotoxic effects. Kruskal–Wallis followed by the Mann–Whitney U test: β-Ti21S, Ti–6Al–4V ELI, and CTR− vs. CTR+, *** *p* < 0.0005.

**Figure 10:**
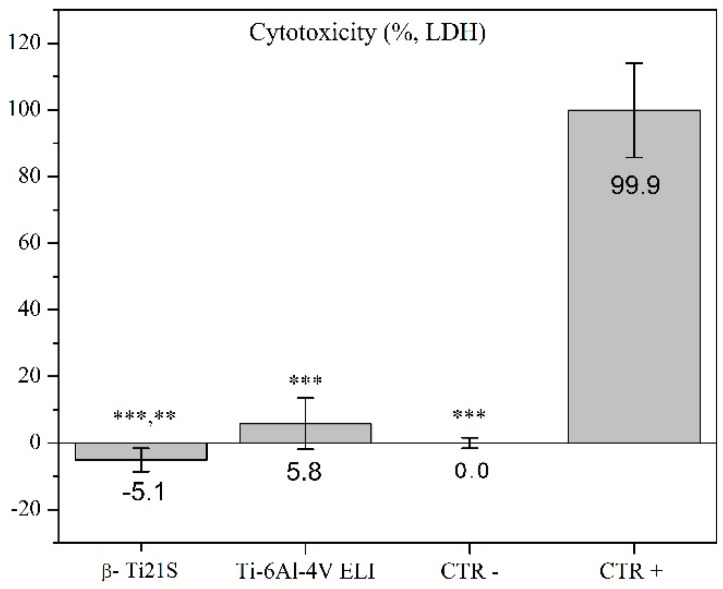
Lactate deydrogenase (LDH) released by the β-Ti21S (test), Ti–6Al–4V ELI (reference), and CTR− (cells without material) and CTR+ (cells with a cytotoxic substance) controls. Kruskal–Wallis followed by the Mann–Whitney U test: ***: β-Ti21S, Ti–6Al–4V ELI and CTR− vs. CTR+, *p* < 0.0005; **: β-Ti21S vs. Ti–6Al–4V ELI and CTR−, *p* < 0.005.

**Figure 11:**
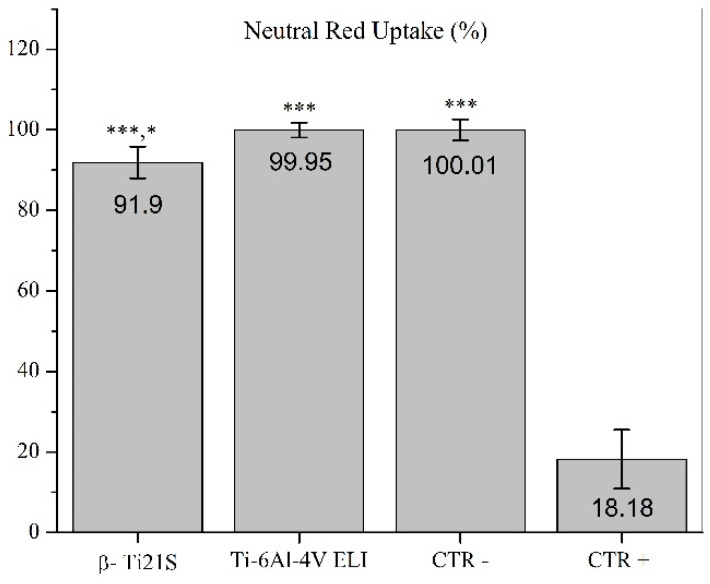
Neutral red uptake quantification of cells exposed to the β-Ti21S (test), Ti–6Al–4V ELI (reference), and CTR− (cells without material) and CTR+ (cells with a cytotoxic substance) controls and expressed as a percentage of CTR−. (Kruskal–Wallis followed by the Mann–Whitney U test. ***: β-Ti21S, Ti–6Al–4V ELI, and CTR− vs. CTR+, *p* < 0.0005. *: β-Ti21S, vs. Ti–6Al–4V ELI and CTR−, *p* < 0.05).

**Figure 12:**
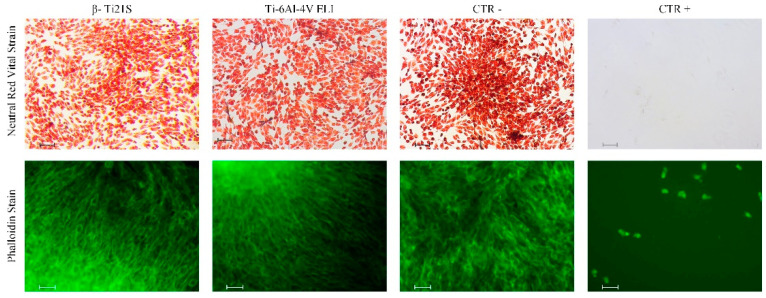
Microscopic images of cells seeded with the β-Ti21S (test), Ti–6Al–4V ELI (reference), and CTR− (cells without material) and CTR+ (cells with a cytotoxic substance) controls. Upper row: cells stained with Neutral Red vital stain (magnification 10×, bar = 100 μm). Lower row: cells stained with Fluorescein Isothiocyanate FITC-conjugate Phalloidin stain (magnification 20×, bar = 10 μm).

The results on the evaluation of MG63 cultured with experimental materials, reference materials, and controls are summarized in Figure 9 (viability test), Figure 10 (LDH), Figure 11 (Neutral Red uptake), and Figure 12 (Phalloidin and Neutral Red stainings). The viability results showed that β-Ti21S (test) and Ti–6Al–4V ELI (reference) samples had higher significant viability than CTR+ (*p* < 0.0005) without any difference in comparison with CTR− and with a percentage of viability over 70%; therefore, no cytotoxicity was detected. Moreover, test and reference materials released a lower amount of LDH than CTR+ (*p* < 0.0005) without any difference in comparison with CTR−; the test sample showed a significantly lower release of LDH even than reference S1 and CTR− (*p* < 0.005). Cytoplasmic membranes of cells exposed to test and reference materials were able to actively uptake Neutral Red vital stain with significantly increased values than CTR+ (*p* < 0.0005); β-Ti21S showed a significantly lower uptake than reference and CTR− (*p* < 0.05). Figure 11 shows microscopic images of Phalloidin and Neutral Red vital stainings, performed to highlight cell morphology in the presence of materials. Phalloidin specifically binds to actin filaments of the cell cytoskeleton [56], while Neutral Red is actively incorporated within cytoplasmic lysosomes only in vital cells [57]. As for CTR−, experimental and reference samples showed that MG63 had a normal elongated morphology without cell detachment, lysis, or cytoplasmic vacuolization; the integrity and permeability of membranes by means of active transport systems were confirmed by the uptake of the Neutral Red vital stain. By contrast, CTR+ was markedly less in number, presented a rounded morphology, and did not take up the vital dye.

The results reported in this paper show that a β-Ti alloy could be successfully produced by additive manufacturing. The achievement of near full density, low defectiveness, and fine full-β microstructure confirm the suitability of the L-PBF parameters. The suppression of martensitic transformation permits the achievement of a metastable β-structure, thus avoiding the inherent brittle structure observed in the as-built state for α + β alloys like Ti–6A–4V. This is made possible by the presence of 15%Mo, which plays a twofold role on the martensitic transformation: first, the critical cooling rate to retain β is decreased, second, the martensitic start temperature is lowered drastically [34].

Mechanical properties are very encouraging compared with those of similar alloys investigated so far in the technical literature (see Table 1). It is worthy to remark that the alloy displays an ultra-low Young’s modulus (52 ± 0.3 GPa), less than half of that of Ti–6Al–4V, widely used for biomedical applications. Moreover, it shows good mechanical strength and excellent ductility without the need of heat treatments. This fact is not of secondary importance considering the costs and the critical issues related to the heat treatment of Ti alloys. The experience of some of the present authors evidenced that oxygen as well as nitrogen and carbon pick-up during vacuum annealing of Ti–6Al–4V may lead to low and poorly reliable mechanical properties. An acceptable anisotropy degree could be determined by compression tests, evidencing slightly higher elastic modulus and lower strength perpendicularly to the building direction. The results are promising, looking to the manufacturing of parts undergoing multiaxial loading. Nevertheless, anisotropy should be also verified under tensile stress conditions, to emphasize any possible influence of the solidification structure, texturing, and defects orientation on mechanical properties.

Considering the detrimental effects of residual stresses in as-built components, those associated to phase transformations are obviously not present in this alloy. On the other hand, thermal stresses can be also reduced owing to the accommodation permitted by the relatively low yield strength and the good ductility of this β-alloy. This is particularly important looking to the future production of larger components than those tested in this paper, as well as to the production of cellular structures, typically used to produce orthopaedic implants.

In vitro cytotoxicity tests results are good, not worse than those of Ti–6Al–4V. The absence of V contributes to the very good biocompatibility properties attested by in vitro cytotoxicity experiments, conducted following the international standard UNI EN ISO 10993-5. In fact, the cell viability was maintained without statistically significant differences with the reference material, without any morphological alterations, and in the absence of the release of cell damage mediators.

Further research is currently underway to investigate the fatigue performance of the present material and its suitability to manufacture cellular lattice structures. Moreover, deeper investigations on biological competence in bioactivity assays using advanced in vitro models and preclinical evaluations of the β-Ti alloy safety and efficacy are mandatory in view of its clinical use for 3D printed implant materials to be used in orthopedic applications with a fully oriented personalized medicine.

## 4. Conclusions

The properties of β-Ti21S produced by laser powder bed fusion were considered in this paper for the first time. Near fully dense samples could be successfully produced from gas atomized commercial powder. The following conclusions can be drawn:

- A fully metastable β structure could be obtained, even despite the rapid solidification;
- Columnar grains formed along the building direction led to texture in <100> orientation. Inside melting pools, the solidification mechanism changed from planar to cellular owing to the establishment of strong constitutional undercooling, caused by the wide alloy freezing range.
- Very interesting mechanical properties could be measured in the as-built state, without any post heat treatment; the Young’s modulus is one of the lowest reported in literature for β-Ti alloys (52 GPa), the mechanical strength is slightly lower than that of Ti–6Al–4V, in line with those of other β-Ti alloys. The high fracture elongation suggests the good strain accommodation capacity and the possibility of limiting distortions.
- Compression tests revealed that texture causes a limited variation (<20%) of the Young’s modulus along different directions.
- Viability results showed that experimental and reference samples had higher significant viability than CTR+, and no cytotoxicity was detected

## Figures and Tables

**Table 1:** Mechanical properties of present β-Ti21S alloy compared with those reported in the literature for additively manufactured and wrought (W) Titanium alloys.

Alloy	σ_y0.2_ (MPa)	UTS (MPa)	E (GPa)	El (%)	Structure	Reference
Ti–6Al–4V ELI ^1,^*	1015	1090	113	10	α′	[39]
Ti–6Al–4V *	990 ± 5	1095 ± 10	110 ± 5	8.1 ± 0.3	α′	[50]
Ti–6Al–4V + 10Mo **	858 ± 16	919 ± 10	73 ± 1	20 ± 2	β	[34]
Ti–7.5Mo *	570	740	70	9.2	α + β	[35]
W–Ti21S 0° ^2^	852 ± 1	867 ± 5	-	16.4 ± 0.0	β	[46]
W–Ti21S 45° ^2^	859 ± 11	884 ± 0.6	-	13.5 ± 0.3	β	[46]
W–Ti21S 90° ^2^	797 ± 8	810 ± 14	-	16.7 ± 0.7	β	[46]
β-Ti21S *	709 ± 6	831 ± 3	52 ± 0.3	21 ± 1.2	β	This study

σ_y0.2_: 0.2% yield stress; UTS: ultimate tensile strength; E: young’s modulus; El: fracture elongation. ^1.^ ELI = extralLow intertitials, ^2.^ W = wrought β-Ti21S alloy samples cut from rolled sheet; the angles highlight the orientation of sample with respect to the rolling direction. * Tensile test sample axis parallel to the building direction. ** Tensile test sample axis perpendicular to the building direction. Where available, mechanical properties are indicated as mean ± standard deviation.
